# Spectroscopic Insights into the Electrochromism of Hexagonal Tungsten Oxides (HTOs)

**DOI:** 10.1021/acsomega.5c12695

**Published:** 2026-01-26

**Authors:** Tao Gao

**Affiliations:** Department of Civil and Environmental Engineering, Norwegian University of Science and Technology (NTNU), NO-7491 Trondheim, Norway

## Abstract

Spectroscopic techniques, including X-ray diffraction (XRD) and Fourier transform infrared (FTIR) spectroscopy, were employed to unravel the microscopic details of proton insertion and extraction in hexagonal tungsten oxides (HTOs), thereby providing new insights into the underlying electrochromic mechanisms. Hexagonal sodium tungsten bronze (Na-WO_3_) was selected as a representative HTO material and prepared hydrothermally. The resulting Na-WO_3_ nanorods had typical diameters of 10–200 nm and lengths of several microns and crystallized in a hexagonal structure (space group *P*6/*mmm*, No. 191) with unit cell parameters *a* = 7.3166(8) Å and *c* = 3.8990(8) Å and elongated along the ⟨001⟩ direction. Proton insertion during the electrochromic (EC) coloration process induced significant changes in both long-range and local structural order, as evidenced by modified lattice parameters (*a* = 7.4192(6) Å, *c* = 7.5440(2) Å) and reduced local symmetry (space group *P*6_3_/*mcm*, No. 193) in the EC colored Na-WO_3_ nanorods. The inserted protons preferentially occupied small trigonal windows rather than larger trigonal cavities, indicating a selective intercalation behavior analogous to that observed in the large hexagonal tunnels. FTIR analysis revealed that proton insertion and extraction in the small trigonal tunnels were closely coupled with the dynamics of water molecules residing in the large hexagonal tunnels. These ionic processes were accompanied by electron transfer, resulting in substantial modifications to the electronic structure of the material. Band gap narrowing (from 2.5 to 1.75 eV) and the emergence of a new near-infrared absorption band (centered at 1155 nm) were observed in the EC colored Na-WO_3_ nanorods, which were attributed to increased free electron densities arising from proton–electron double charge injection.

## Graphical abstract

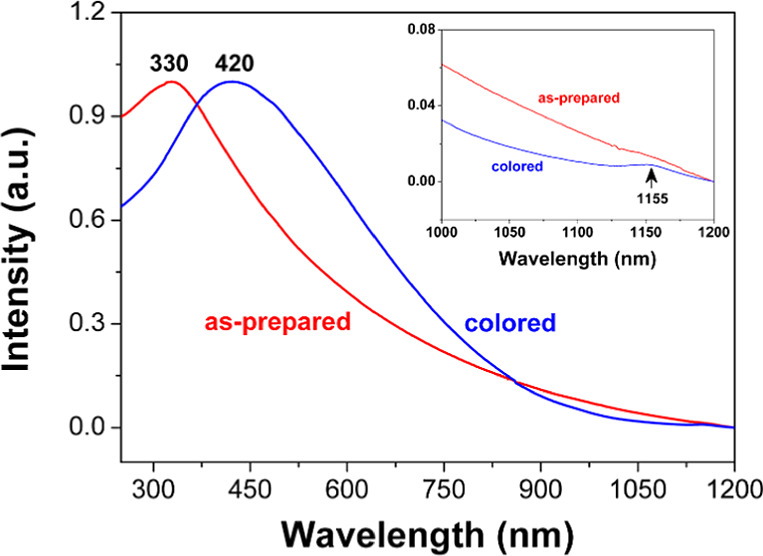

## Introduction

Over the past decades, there has been an increasing interest in dynamic glazing technologies, also known as smart windows.^1–4^ The primary advantage of smart windows is their capability to control the radiant energy (both visible light and heat) transmitted through the window aperture, hence providing opportunities to maximize building energy efficiency by reducing a certain amount of HVAC (heating, ventilation, and air conditioning) loads.^5^ In addition, smart windows offer other technical benefits such as reduced glare for a better visual comfort and increased architectural aesthetics and flexibility through reduction or elimination of shading devices. Smart windows are also very promising in the automotive industry, especially in electric vehicles, where both user comfort and energy efficiency (i.e., mileage) are of great importance.

A smart window is in principle an optical switching device integrated in or attached to a window glazing and offers adjustable control over the transmitted radiant energy.^1^ Apparently, the key to smart windows is a material or system that can change reversibly its optical properties (transmittance, reflectance, etc.) under certain conditions. So far, various actively modulated or passively responsive smart window technologies have been developed,^3–5^ including chromogenic materials (e.g., electrochromic (EC), thermochromic (TC), photochromic (PC), and gasochromic (GC) materials), suspended particle (SP) devices, polymer dispersed liquid crystal (PDLC) films, and reversible metal electrodeposition (RMED) devices. Among these, EC smart windows represent probably the most technologically mature dynamic glazing solution and have achieved a modest level of application in the high-end market.^5^ However, widespread market adoption of EC smart windows has been slow and hindered by several drawbacks related to cost, durability, and functionality—factors that also affect other smart window technologies. Apparently, the success of smart windows depends on not only the potential benefit that will be realized by the end users, but also a sophisticated design and selection of materials to make the devices durable and affordable.^6^ In this regard, a firm understanding of the underlying mechanisms and microscopic details of the employed materials or systems during the electrochemical cycles will significantly impact the progress.

We have previously reported hexagonal sodium tungsten bronze (namely, Na-WO_3_) nanorods with interesting structural and chromogenic properties.^7,8^ Na-WO_3_ belongs to the family of hexagonal tungsten oxides (HTOs),^9,10^ which are characterized by their unique tunnel structure featuring one-dimensional hexagonal and trigonal nanochannels within a framework of vertex-shared WO_6_ octahedra. The structural openness of HTOs makes them very promising for applications in electrochemical devices,^9,11^ where the mass transport can be greatly facilitated, typically resulting in fast kinetics that are essential for high-performance electrochemical devices such as EC smart windows.^8,12,13^ This, on the other hand, may raise a compelling question regarding the inserted ions (e.g., H^+^ or Li^+^), given that the HTOs are typically stabilized through the intercalation of various cations, such as Li^+^, NH_4_^+^, Na^+^, and K^+^, within the large hexagonal tunnels.^10,11^ In this regard, different intercalation thermodynamics and kinetics may apply, as the intercalated ions typically occupy distinct tunnel sites depending on factors such as their ionic sizes or intermolecular bonding characteristics.^14–20^ For example, Chen et al.^17^ reported previously that NH_4_^+^ ion preintercalated HTOs can effectively enhance NH_4_^+^ adsorption energy and reduce its diffusion energy barrier in the hexagonal tunnels. For smaller ions such as Li^+^, Hibino et al.^18^ found that the inserted Li^+^ ions are more stable in trigonal tunnels than at other sites; in addition, Li^+^ ions begin to occupy hexagonal tunnels before the trigonal ones are fully filled, and they leave the trigonal tunnels only after those in the hexagonal tunnels are nearly depleted.^19^ Our previous work also indicated that the small trigonal tunnels are energetically more favorable for the inserted protons,^7,8^ as the repulsive effect of stabilizing tunnel cations may further discourage proton occupation in the large hexagonal tunnels. This was in agreement with the previous report on mixed hexagonal lithium potassium tungsten bronzes,^20^ Li_*x*_K_*y*_WO_3_ (*x* ≤ 0.66, *y* ≤ 0.33), where the smaller Li^+^ ions occupied sites in the trigonal tunnels. Interestingly, Han et al.^19^ found that the presence of tunnel cations (i.e., NH_4_^+^ ions) has little impact on the Li^+^ ion insertion properties, apart from a slight kinetic effect. Similar observations were also reported in our previous studies on the electrochemical properties of Na-WO_3_ nanorods;^7,8^ however, our findings revealed that proton insertion induces significant structural evolutions,^8^ going beyond the minor lattice parameter changes described in earlier reports.^18–20^

From the perspective of structure–property relationships, this work aims to unravel the microscopic details of ion insertion and extraction in HTOs during the electrochemical cycles. These processes are found to be accompanied by substantial structural evolutions and also give rise to novel physical properties that remain largely unexploited. In particular, spectroscopic techniques,^21^ such as X-ray diffraction (XRD) and Fourier transform infrared (FTIR) spectroscopy, which are sensitive to long-range and local structural order, respectively, can be conveniently employed to monitor in situ the structural evolutions during the bleaching process, thereby shedding light on the underlying electrochromic mechanisms.

## Experimental Procedures

### Chemicals and Materials

Reagent-grade sulfuric acid (H_2_SO_4_, 96%), sodium tungstate dihydrate (Na_2_WO_4_·2H_2_O), oxalic acid (H_2_C_2_O_4_), and sodium sulfate (Na_2_SO_4_) were purchased from Sigma-Aldrich Co. and used as received. Distilled water was used throughout the experiment.

### Synthesis of Hexagonal Na-WO_3_

In a typical synthesis,^7^ 11.4 g Na_2_WO_4_·2H_2_O was dissolved in 150 mL water, and to this solution 15 mL concentrated H_2_SO_4_ was added dropwise under constant stirring. The formed precipitation was separated from the reaction solution by centrifugation, washed four times with distilled water (by filtration), and finally dissolved in 300 mL oxalic acid aqueous solution (0.4 M). The obtained colorless, transparent tungstate–oxalate complex (TOC) solution was used as precursors for the hydrothermal synthesis of hexagonal Na-WO_3_ in this work.

For a typical hydrothermal synthesis, 30 mL of TOC solution was transferred into a Teflon-lined autoclave (capacity 40 mL). After adding 1 g of Na_2_SO_4_, the autoclave was sealed and heated at 180 °C for 24 h. After the reaction, the autoclave was cooled down to room temperature by tap water. The obtained white sediments were filtered, washed with water to remove the residual ions/chemicals, and then dried at 60 °C for 5 h to give the as-prepared Na-WO_3_ materials.

### Characterization

Phase analysis was based on powder X-ray diffraction (XRD) data collected on a Siemens D5000 Powder X-ray Diffractometer (with Cu Kα_1_ radiation) equipped with an automatic antiscatter slit and a scintillation detector. The XRD data were measured between 5 and 70° in 2θ in reflection geometry. The obtained XRD patterns were also analyzed by the Rietveld method.^7^ The morphology and chemical composition of the as-prepared materials were investigated by field-emission scanning electron microscopy (SEM, Zeiss Supra 55VP) and transmission electron microscopy (TEM, JEOL JEM-2010), both equipped with energy-dispersive X-ray spectrometers (EDS). Attenuated total reflectance (ATR) Fourier transform infrared (FTIR) spectra were recorded on a Nicolet 8700 FTIR Spectrometer (Thermo Scientific) with a spectral resolution of 2 cm^–1^. Perkin-Elmer LAMBDA 1050 UV/vis/NIR spectrophotometer was also used to measure the optical properties of the obtained materials and samples. Samples for absorbance measurement were prepared by ultrasonically dispersing a small amount of the as-prepared materials in water to form a diluted suspension. Electrochemical properties were characterized on an Autolab electrochemical workstation (PGSTAT302N). A three-electrode electrochemical cell was prepared, where Pt wire, Na-WO_3_ on ITO glass, Ag/AgCl electrode, and 1 M H_2_SO_4_ aqueous solution act as counter electrode, working electrode, reference electrode, and electrolyte, respectively. All measurements were performed at room temperature.

In situ XRD and ATR–FTIR experiments were conducted to monitor the structural changes during the bleaching process under ambient air exposure. The above-mentioned XRD and FTIR experiments were repeated at defined time intervals (as detailed in the main text). To prevent potential photochromic effects,^7^ the samples were covered with aluminum foils during air exposure, which were then removed for the XRD and FTIR measurements.

## Results and Discussion

Figure 1a shows a typical XRD pattern of the as-prepared materials. The XRD reflections are sharp and intensive, indicating that the as-prepared materials are well crystallized. In addition, all the observed XRD reflections can be indexed on the basis of a hexagonal sodium tungsten bronze Na-WO_3_ with unit cell dimensions of *a* = 7.331 and *c* = 3.891 Å (JCPDF 81-0577). It indicates that a HTO phase stabilized by Na^+^ ions has been produced.^7,12,22^ It is important to point out that there are two distinguished tunnel types with different dimensions in HTOs, namely, large hexagonal tunnels and small trigonal tunnels: the large hexagonal tunnels are occupied by tunnel species such as alkaline metal ions (e.g., Li^+^, Na^+^, and K^+^), which are essential to stabilize the hexagonal structure;^23^ the small trigonal tunnels are usually empty, but may host small ions such as H^+^ or Li^+^ during different physical or chemical processes.^8,18–20^

**Figure 1:**
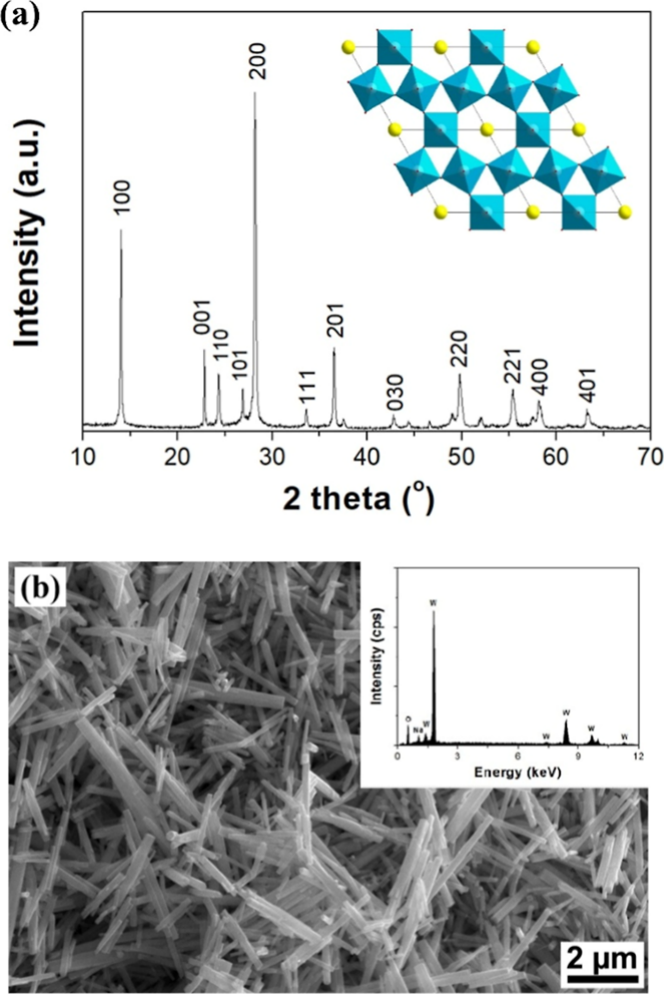
(a) XRD pattern of the as-prepared materials. Miller indices are given on the basis of a hexagonal sodium tungsten bronze Na-WO_3_ (JCPDF 81-0577). Inset shows a polyhedral representation of the hexagonal Na-WO_3_ viewed along the *c*-axis; tunnel species (Na^+^ ions and/or water molecules) are represented by yellow spheres. (b) SEM micrograph of the as-prepared hexagonal Na-WO_3_. Inset shows the corresponding EDS results.

Further structural characterization has been performed by scanning electron microscopy (SEM) together with energy-dispersive X-ray spectroscopy (EDS). The SEM analyses (Figure 1b) indicate that the as-prepared materials are nanorods with typical diameters of about 20–200 nm and lengths up to several micrometers. EDS data reveal that these nanorods are composed of Na, W, and O (probably also H that is not detectable by EDS) with an average Na/W element ratio of about 1:5.5, giving a mean chemical composition of Na_0.18_WO_3_ for the as-prepared materials. It is worth noting that alkali metal ion intercalated HTOs can also be formulated as M_*x*_WO_3+*x*/2_·*y*H_2_O, where *x* = 0.17–0.25 for M = Na.^22^ In this work, the stoichiometry of the as-prepared materials can be given as Na_0.18_WO_3.09_·*y*H_2_O, where the water content y is about 0.5 according to the corresponding thermogravimetric analysis. For simplicity, Na-WO_3_ will be used hereafter to represent the as-prepared Na_0.18_WO_3.09_·0.5H_2_O nanorods in this work.

Na-WO_3_ nanorods have been reported to exhibit a predominant cathodic electrochromism involving a double insertion of protons (or Li^+^ ions) and electrons^8^

1 $$Na‐WO_{3}+x\left(H^{+}+e^{-}\right)↔Na‐H_{x}W_{\left(1-x\right)}^{6+}W_{x}^{5+}O_{3},\left(0<x<1\right)$$

where the electrochemical reduction of tungsten ions (i.e., W^6+^ + e^–^ ↔ W^5+^) leads to dark blue coloration,^12^ and proton insertion helps maintain charge neutrality in the materials. As shown in Figure 2, the Na-WO_3_ nanorods after EC coloration (hereafter denoted as EC colored Na-WO_3_ nanorods) present a distinctive dark blue appearance when viewed from the top, while showing a striking red-brown metallic reflection at tilted angles. This contrasts sharply with the original Na-WO_3_ nanorod films, which appear white with a slight yellow tint regardless of viewing angles. Although the coloration process is reversible,^8,12^ the subsequent bleaching process is notably slower, especially for thicker films such as those shown in Figure 2). The distinct ion insertion and extraction behaviors suggest that the Na-WO_3_ nanorods might undergo substantial structural modifications during the electrochemical cycles. This has been confirmed by the corresponding X-ray diffraction (XRD) investigations.

**Figure 2:**
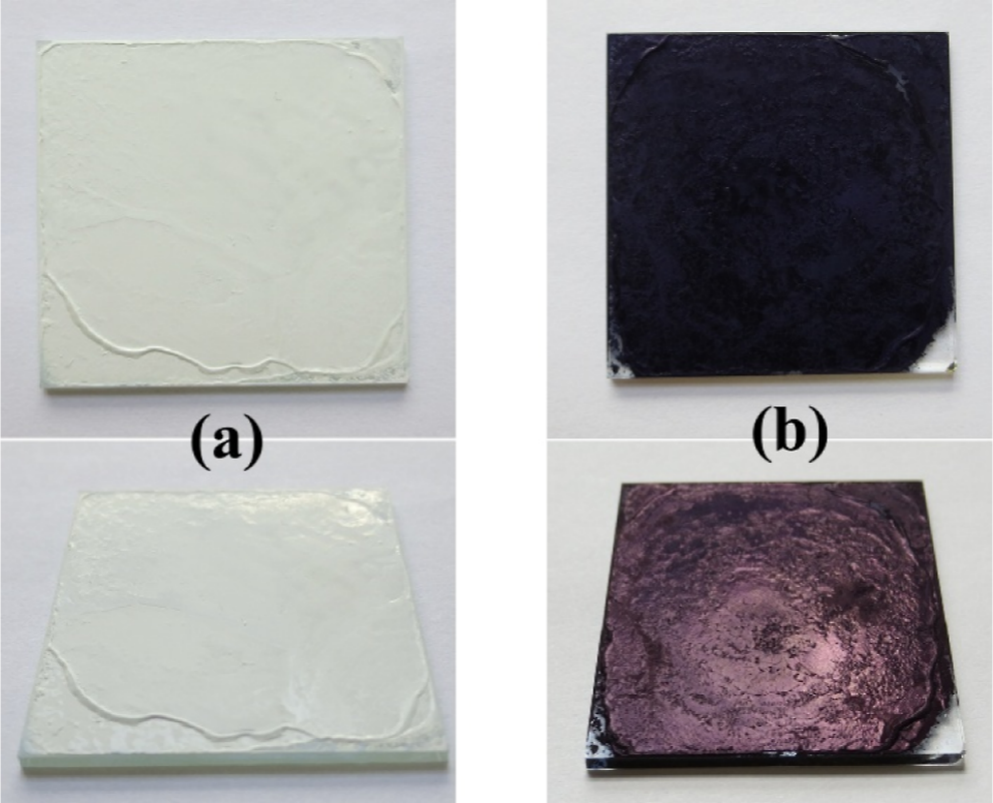
Photographs of Na-WO_3_ nanorod films before (a) and after (b) the electrochromic coloration. Top images: normal incidence (90° to the film surface); bottom images: tilted view at about 45°.

Rietveld refinement of the acquired XRD data is performed to elucidate changes in the long-range structural order. Crystallographic data and details in the data collection and structure refinement are reported in Table 1. The final observed, calculated, and difference patterns are shown in Figure 3. For comparison, the XRD pattern of the pristine Na-WO_3_ nanorods is also included during the refinement. It is worth noting that refining the XRD pattern of the EC-colored Na-WO_3_ nanorods by using the original hexagonal settings (i.e., space group *P*6/*mmm*, *a* = 7.3166(8) Å, and *c* = 3.8990(8) Å) results in nonindexed reflections, suggesting that the EC colored Na-WO_3_ nanorods might be structurally different from the original phase. Although accurately determining the crystallographic details of the EC-colored Na-WO_3_ nanorods is currently challenging due to the relatively low quality of the XRD data and the phase instability (discussed later in this work), the preliminary refinement results indicate that the EC colored Na-WO_3_ nanorods can be indexed on the basis of a hexagonal unit cell (space group *P*6_3_/*mcm*, No. 193) with lattice parameters of *a* = 7.4192(6) Å and *c* = 7.5440(2) Å. It is important to point out that this new hexagonal phase maintains the same tunnel structure as the original hexagonal phase, except for slight atomic position shifts, presumably to accommodate the inserted protons. The observed symmetry reduction from *P*6/*mmm* (No. 191) to *P*6_3_/*mcm* (No. 193), although minor, further reveals that proton insertion in HTOs (i.e., Na-WO_3_ in this work) induces both local- and long-range structural modifications. This may be better understood by examining the WO_6_ distortions associated with the proton insertion.

**Table 1:** Results and Relevant Information for the Rietveld Refinement of Na-WO_3_ Nanorods^a^

phase	Na-WO_3_	Na-HWO_3_ ^b^
temperature/K	298	298
wavelength/Å	1.54056	1.54056
pattern range/deg	10–70	10–70
space group	*P*6/*mmm* (No. 191)	*P*6_3_/*mcm* (No. 193)
*a*/Å	7.3166(8)	7.4192(6)
*c*/Å	3.8990(8)	7.5440(2)
*V*/Å^3^	180.7659(3)	359.6261(2)
*Z*	1	2
no. reflections	26	37
no. refined parameters	16	14
*R*_wp_	0.11	0.12
*R*_p_	0.08	0.09

a Calculated standard deviations are in parentheses; the *R* factors are defined in ref 7.

b H atoms are not included in the refinement.

**Figure 3:**
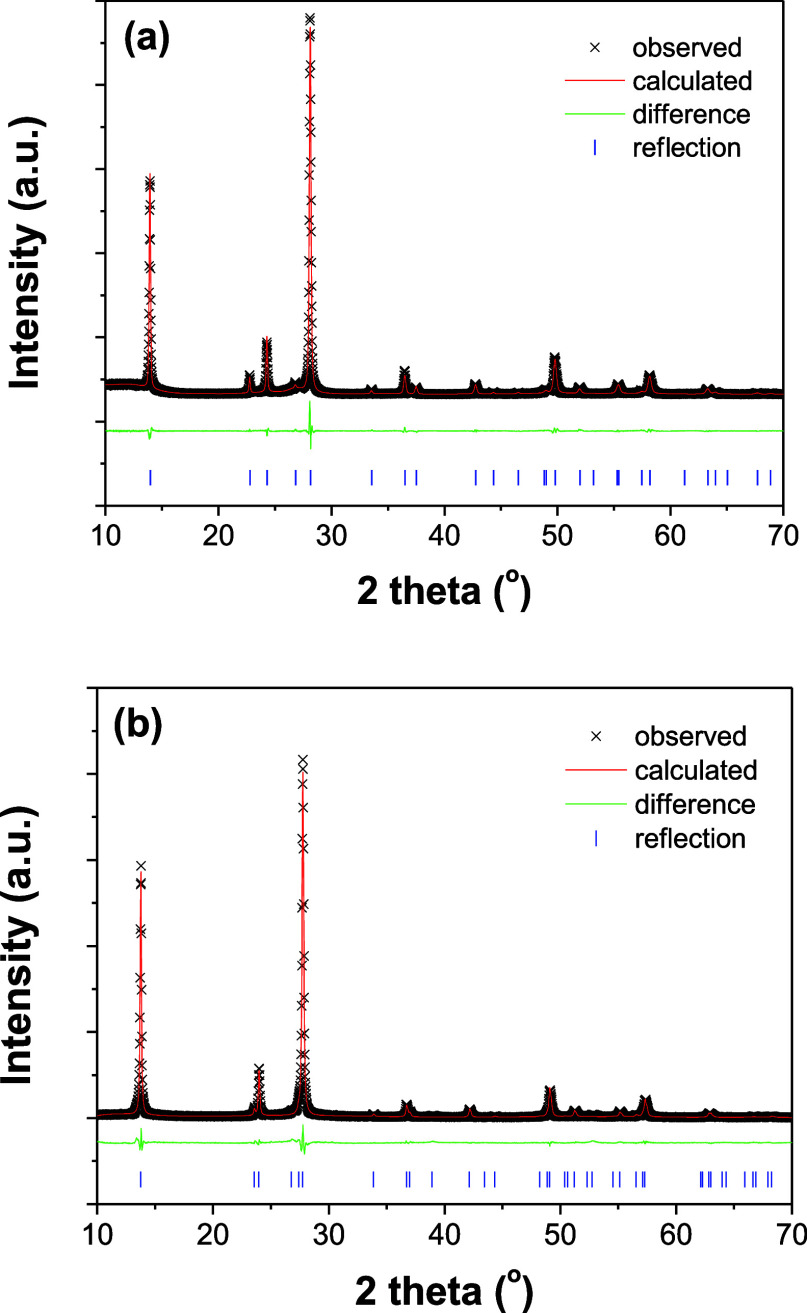
Final results of the Rietveld refinement of the structure of Na-WO_3_ nanorods before (a) and after (b) the electrochromic coloration. Observed and calculated profiles are shown by crosses and solid lines, respectively. The positions of the reflections are indicated at the bottom. X-ray wavelength: 1.54056 Å.

Table 2 presents selected interatomic distances within a representative WO_6_ octahedron from both phases for comparison (see, for example, Figure 4). The original Na-WO_3_ has a simple hexagonal structure in the space group of *P*6/*mmm*, in which the WO_6_ octahedron is nearly perfect: 4 short W–O1 bonds (about 1.901 Å) in the basal plane and 2 slightly longer W–O2 bonds (about 1.944 Å) in the axial plane; the O2 atoms and the W atoms alternate along the *c* direction. During the EC coloration process, local structural modifications are required to accommodate the intercalated protons. As a result, the WO_6_ octahedron is significantly distorted: Each W atom now has two long W–O1 bonds (about 2.095 Å) and two short W–O1 bonds (about 1.805 Å) in the *ab* plane, and two short W–O2 bonds of about 1.912 Å along the *c* axis (see Figure 4). The fact that W atoms are displaced toward the edge of the WO_6_ octahedron indicates that the intercalated protons are most likely bonded to the O1 atoms in the *ab* planes instead of the O2 atoms along the *c* axis. This can be attributed to the W–O bond length and/or strength changes due to the attached protons (i.e., W–O···H). In addition, the displacement of W and O1 atoms in the *ab* planes results in the two W–O2 bonds along the *c* axis having the same length. This is in harmony with the hexagonal symmetry *P*6_3_/*mcm*, where the mirror plane is perpendicular to the *c* axis so that W–O bond lengths along the *c* axis should be equal.

**Table 2:** Selected Interatomic Distances (Å) in WO_6_ Octahedron in Na-WO_3_ Nanorods

as-prepared Na-WO_3_	
W–O1	1.901 (×4)
W–O2	1.944 (×2)
EC Colored Na-WO_3_	
W–O1	2.095 (×2)
W–O1	1.805 (×2)
W–O2	1.912 (×2)

**Figure 4:**
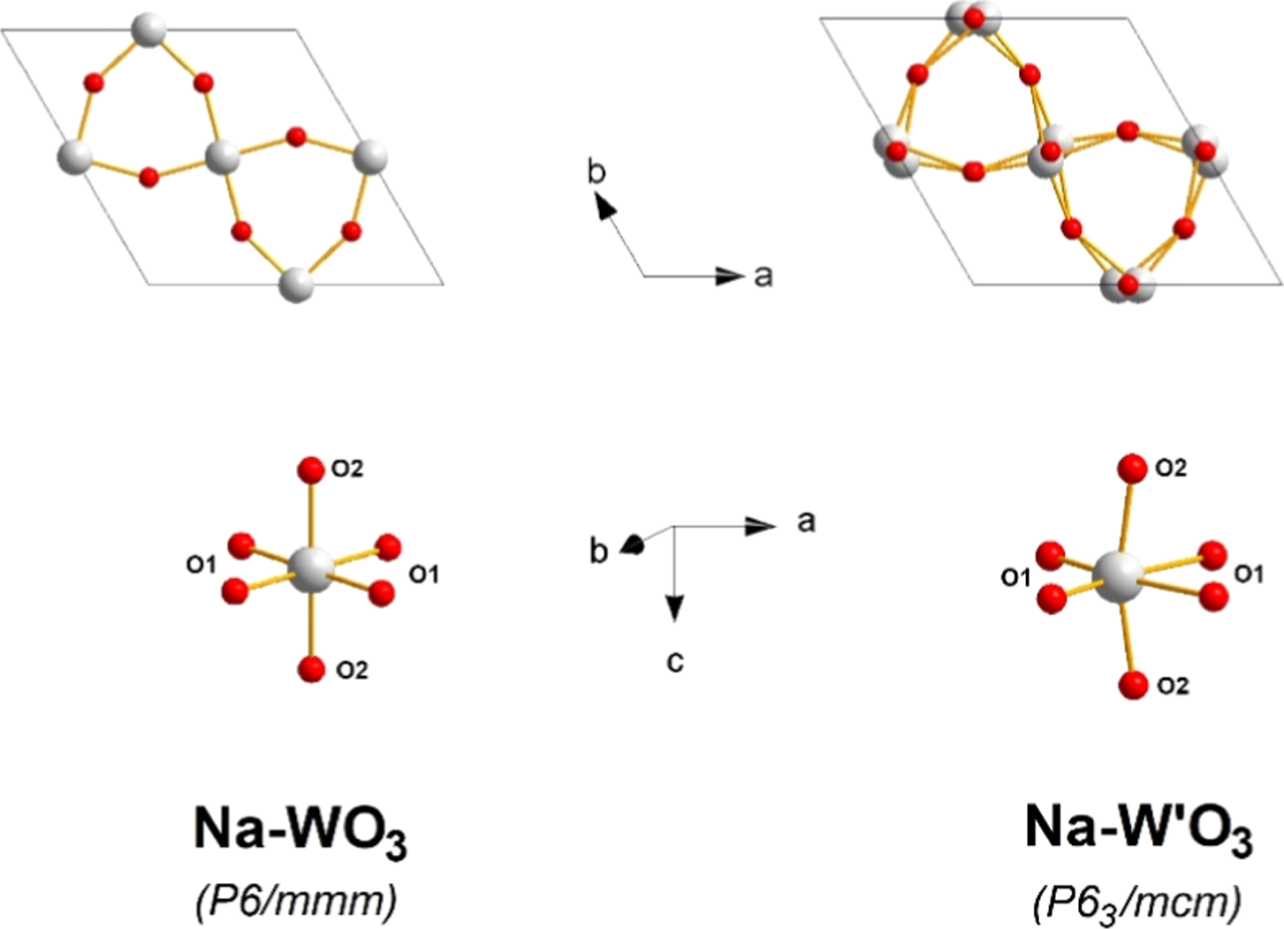
Structural distortion of hexagonal Na-WO_3_ during the EC coloration process. W' stands for the W atoms after coloration. Tunnel species and inserted protons are omitted for clarity.

Since the inserted protons bond to oxygen atoms and occupy the tunnel positions, an intriguing question immediately arises regarding the specific locations of the protons within the hexagonal lattice. A closer look at the open tunnels in HTOs indicates the presence of two distinctive tunnel positions,^24^ namely, A- and B-site, according to their local chemical environment, as shown in Figure 5. For the large hexagonal tunnels (Figure 5a), the A_H_-site at 1*a* (0, 0, 0) has six nearest neighbor O1 atoms with an A_H_–O1 bond length of about 2.665 Å. Similarly, the B_H_-site at 1b (0, 0, 0.5) has six nearest neighbor O2 atoms with a longer B_H_–O2 bond length of about 3.663 Å. These features directly give rise to the ion-exchange selectivity in HTOs.^14–16^ For example, smaller metal cations such as Na^+^ ions (Shannon radius 1.02 Å for 6-fold coordination^25^) usually occupy the A_H_-sites,^24^ whereas larger cations such as K^+^ ions (Shannon radius 1.38 Å for 6-fold coordination^25^) or NH_4_^+^ ions (Shannon radius 1.48 Å for 6-fold coordination^25^) are usually located at the B_H_-sites.^17,20,24^ Tunnel water molecules are also found at the B_H_-sites due to their relatively large dimensions (radius of about 1.34 Å^26^), as revealed by nuclear magnetic resonance spectroscopy^15,16^ and Rietveld refinement.^7,22^

**Figure 5:**
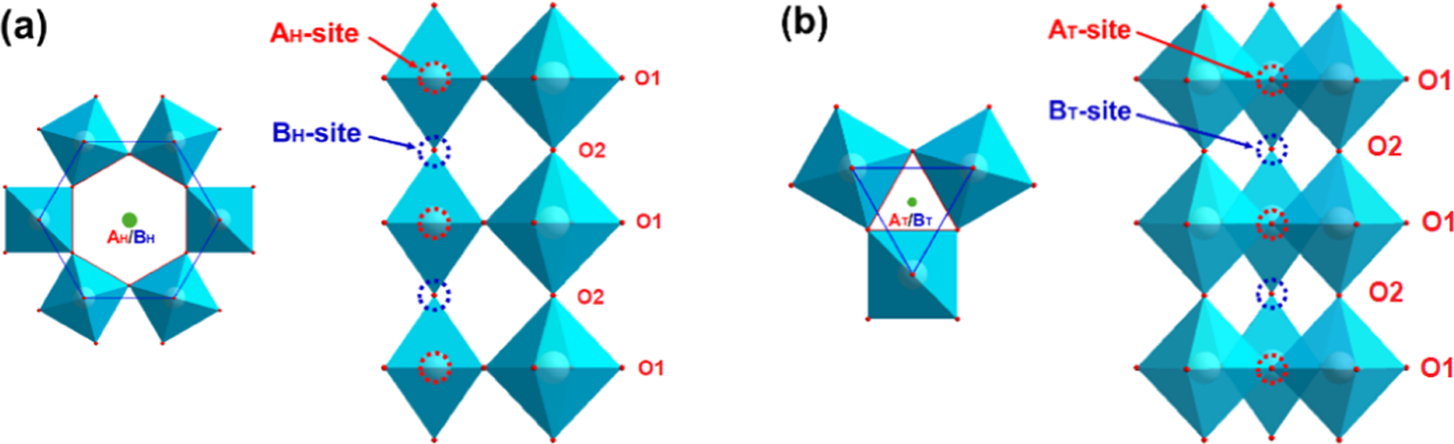
Top and side view of the tunnel structure in hexagonal Na-WO_3_. The two tunnel sites A and B in (a) hexagonal and (b) trigonal tunnels are marked with subscript *H* and *T*, respectively. Tunnel species are removed for clarity.

A similar intercalation chemistry is likely to occur within the small trigonal tunnels (Figure 5b), where the tunnel positions, i.e., A_T_- and B_T_-site, appear to be analogous to their counterparties in the large hexagonal tunnels. For example, the A_T_-site has three nearest neighbor O1 atoms with an A_T_–O1 bond length of about 1.56 Å, whereas the B_T_-site has three nearest neighbor O2 atoms with a B_T_–O2 bond length of about 2.11 Å. Previously, Hibino et al.^18^ calculated the electron energy level in Li^+^ ion intercalated HTOs and concluded that the inserted Li^+^ ions prefer the trigonal cavities, i.e., the B_T_-sites, before they occupy the hexagonal windows (i.e., A_H_-sites) and hexagonal cavities (i.e., B_H_-sites) at higher intercalation levels. It appears that the possibility of Li^+^ ions occupying the A_T_-sites might be overlooked, even though these trigonal windows may well accommodate Li^+^ ions due to their comparable dimensions (approximately 0.78 Å, compared to the Shannon radius of Li^+^: 0.76 Å for 6-fold and 0.59 Å for 4-fold coordination^25^). In this work, since the large hexagonal tunnels in Na-WO_3_ are already occupied by tunnel species such as Na^+^ ions (at A_H_-sites) and water molecules (at B_H_-sites),^7,22^ the inserted protons would be accommodated in the small trigonal tunnels. Furthermore, the inserted protons would occupy the A_T_-sites and bond to the O1 atoms, resulting in significant WO_6_ distortions within the *ab* planes (see Figure 4). However, given the extremely small size of protons, the possibility that inserted protons may be accommodated in the trigonal cavities (i.e., B_T_-sites) or other tunnel positions cannot be totally ruled out,^18,19^ especially at higher intercalation levels. In addition, the large hexagonal tunnels and small triagonal tunnels in HTOs are interconnected,^18^ making it very challenging to obtain a complete microscopic picture of the inserted protons. Further investigations may still be required.

In contrast to proton insertion, the proton extraction corresponds to the bleaching process that restores the EC colored Na-WO_3_ nanorods to their original state. Interestingly, the bleaching process can also be performed in air, although a different oxidation scheme than that in solutions may apply

2 $$W^{5+}-e^{-}↔W^{6+}$$

3 $$O_{2}+{4H}^{+}+{4e}^{-}↔{2H}_{2}O$$

where molecular oxygen is reduced to water due to the availability of protons within the structure. During the bleaching process, these protons diffuse from the small trigonal tunnels, while the resulting water molecules migrate into the larger hexagonal tunnels. It appears that the proton insertion and extraction in the small trigonal tunnels are closely associated with the dynamics of water molecules in the large hexagonal tunnels.^7,8^ The ability to carry out the bleaching process under ambient conditions makes it suitable for in situ or online monitoring using spectroscopic techniques such as XRD and FTIR, which are known for their sensitivity to changes in long-range and local structural orders.^21^

As shown in Figure 6, in situ XRD results clearly revealed a phase transformation during the bleaching process of the EC colored Na-WO_3_ nanorods under air exposure. In addition, no obvious changes in lattice parameter were observed for either the colored or the bleached Na-WO_3_ nanorods during the process. This sharply contrasts with previous reports on Li^+^ ion insertion and extraction in HTOs,^18,19^ where the lattice parameters *a* and *c* increase and decrease, respectively, during the Li^+^ ion insertion, with the opposite trend being reported during the Li^+^ ion extraction process. It is worth pointing out that, as shown in Figure 6b, the (020) crystallographic planes in the EC-colored Na-WO_3_ nanorods evolve into the (200) planes in the bleached phase. Since these planes intersect only the trigonal tunnels in the hexagonal lattice, the observed changes in the corresponding XRD intensities further reveal that the phase transformation during the proton insertion and extraction mostly involves the species confined within the small trigonal tunnels.

**Figure 6:**
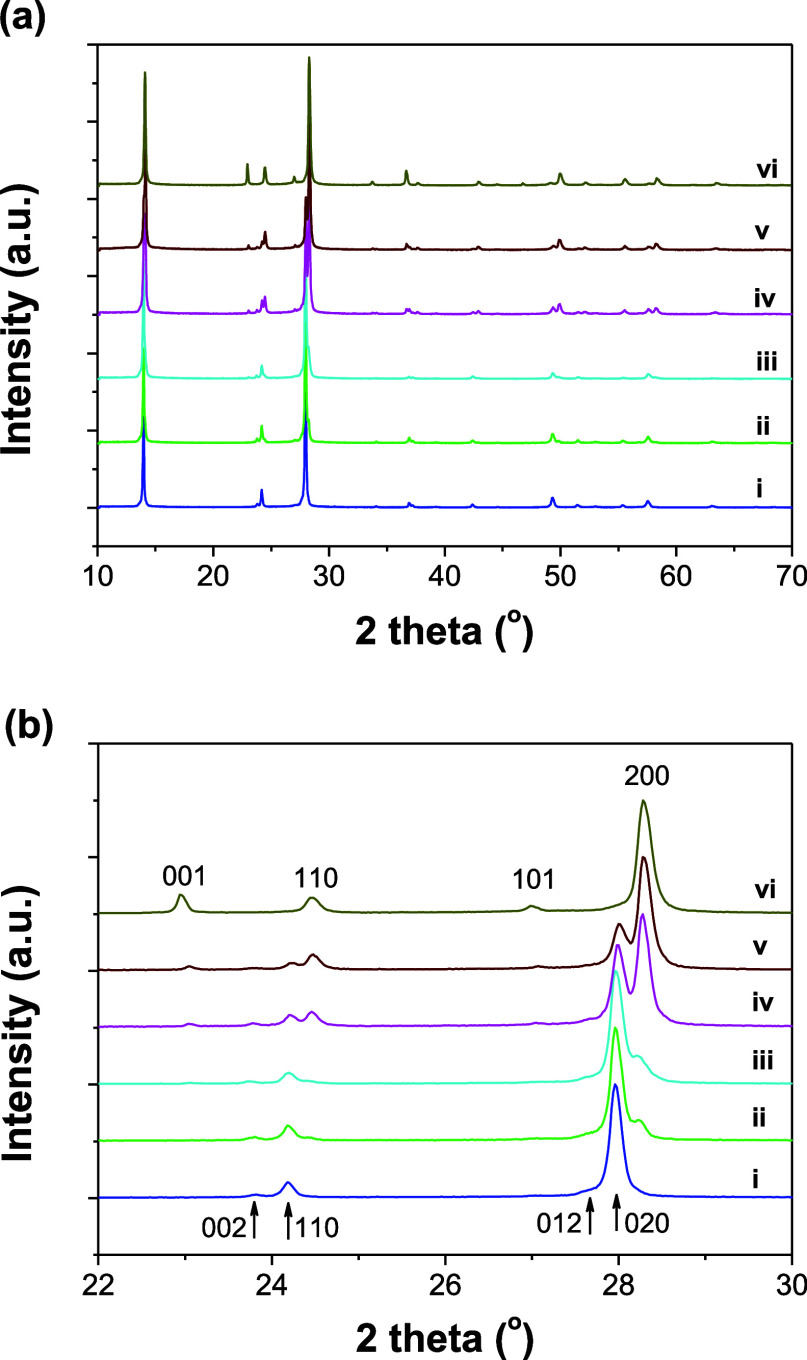
XRD patterns of the EC colored Na-WO_3_ nanorods (i) and those stored in air for (ii) 1 h, (iii) 3 h, (iv) 12 h, (v) 24 h, and (vi) 48 h. In panel b, the Miller indices below and above correspond to the *P*6_3_/*mcm* and *P*6/*mmm* unit cells, respectively. The XRD curves are shifted vertically for clarity.

As shown in Figure 7, in situ FTIR results provided additional insight into the phase transformation occurring in the bleaching process. A notable feature in Figure 7 is the reappearance of vibrational modes associated with tunnel water molecules. For example, the intensities of the water stretching vibrations (3604 and 3545 cm^–1^) and bending vibrations (1622 and 1600 cm^–1^)^7^ increase with air exposure time, indicating the restoration of tunnel water molecules, consistent with the proposed bleaching mechanism (eqs 2 and 3). Another notable feature in Figure 7 is the significant suppression or even disappearance of FTIR bands associated with the EC-colored Na-WO_3_ nanorods. After all, both the colored and the bleached materials exhibit a similar hexagonal structure (i.e., *P*6/*mmm* vs *P*6_3_/*mcm*), for which comparable selection rules should apply to the corresponding lattice vibrations.^27^ Hence, the distinct FTIR responses observed here are likely to reflect the changes in the electronic structure of the Na-WO_3_ nanorods during the proton insertion and extraction. We suggest that the proton–electron double charge injection in Na-WO_3_ nanorods would substantially increase their free electron densities, resulting in strong electron–photon interactions that may significantly dampen the corresponding lattice vibration modes.^28^ This typically leads to broadened, weakened, or even fully suppressed phonon features in the vibrational spectrum. In addition, the increased free electron density may contribute to the pronounced metallic reflections observed in the EC colored Na-WO_3_ nanorod films (see Figure 2b).

**Figure 7:**
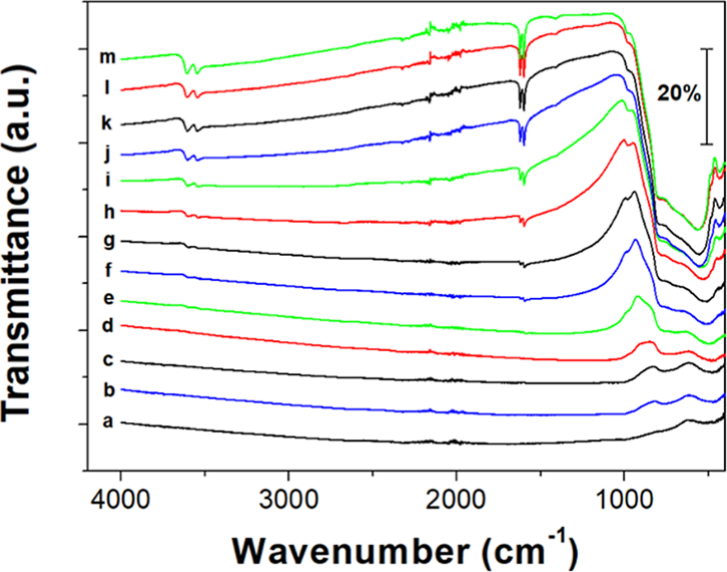
FTIR spectra of EC colored Na-WO_3_ nanorods (a) and those after air exposure for (b) 10 min, (c) 30 min, (d) 1 h, (e) 2 h, (f) 3 h, (g) 4.5 h, (h) 6 h, (i) 7 h, (j) 8 h, (k) 9 h, (l) 10 h, and (m) 24 h. The noise around 2000 cm^–1^ is due to the ATR diamond crystal. The curves are shifted vertically for clarity.

Figure 8 shows the UV–vis optical absorption spectrum of the as-prepared Na-WO_3_ nanorods, together with that of the EC-colored samples for comparison. Notably, the broad absorption band around 330 nm shifts to approximately 420 nm after the EC coloration, indicating a substantial change in the electronic structure of the Na-WO_3_ nanorods as a result of proton–electron double charge injection. As shown in Figure 9, the optical band gap energy of the as-prepared Na-WO_3_ nanorods, estimated by using a direct-allowed electronic transition model,^29^ is about 2.5 eV; after EC coloration, the band gap narrows significantly to around 1.75 eV. Although the detailed mechanism underlying the observed band gap narrowing in Na-WO_3_ nanorods remains under investigation, it is likely associated with band gap renormalization induced by the increased free electron density resulting from proton–electron double charge injection. Previously, Lee et al.^24^ investigated the intercalation chemistry of HTOs using first-principles calculations and found that intercalated alkali-metal ions such as Na^+^ and K^+^ promote an upshift of the Fermi level to the conduction band, suggesting that varying the concentration of intercalated alkali-metal ions might effectively regulate charge density and charge transport in the resulting HTOs. Our findings appear consistent with this interpretation, as well as with previous reports on hexagonal Li_*x*_K_*y*_WO_3_ (*x* ≤ 0.66, *y* ≤ 0.33), where increasing the Li^+^ ion concentration yielded a substantial enhancement in electrical conductivity of the material.^20^

**Figure 8:**
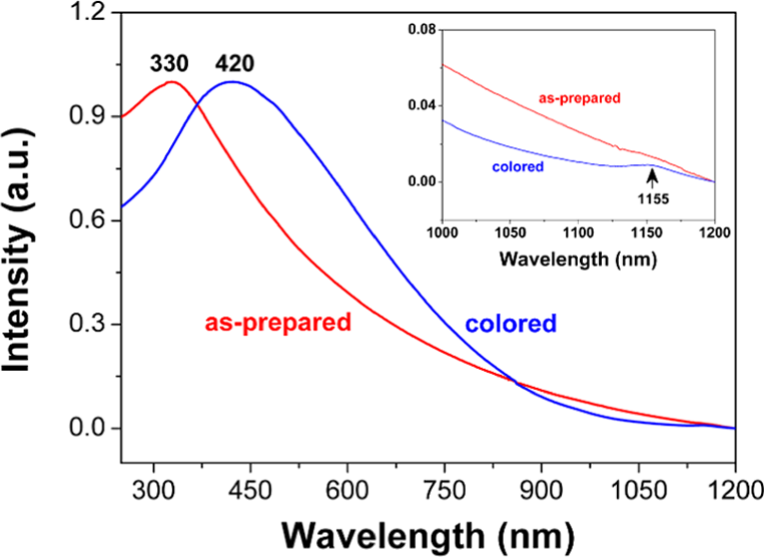
UV–vis absorption of Na-WO_3_ nanorods before and after the EC coloration process.

**Figure 9:**
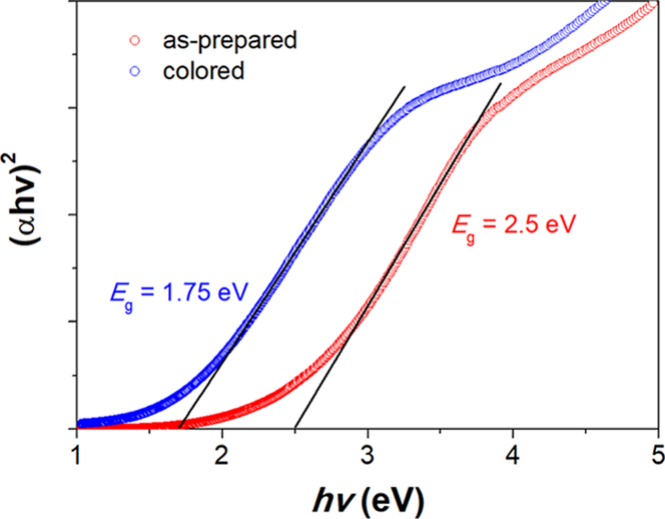
Band gap energy *E*_g_ of Na-WO_3_ nanorods before and after the EC coloration process, determined based on a direct-allowed electron transition model ((α*hv*)^2^ vs *hv*).

The increased charge density resulting from proton–electron double charge injection may also account for the emergence of a new absorption band in the near-infrared (NIR) region, centered around 1155 nm (inset of Figure 8), which is markedly enhanced after the coloration process. This NIR absorption can be attributed to localized surface plasmon resonance (LSPR),^3,30–32^ a phenomenon typically observed in metallic nanoparticles or heavily doped semiconductor nanocrystals with high free electron densities.^30^ Apparently, the small sizes and high crystallinity of Na-WO_3_ nanorods are among the key factors contributing to the observed LSPR band,^32^ especially after the EC coloration where the charge density of the material is significantly increased due to the proton–electron double charge injection. It is worth noting that this LSPR band is active in the NIR region, which is particularly promising for the development of dual-band EC structures or devices capable of independently modulating visible and NIR solar radiation.^3,31^

## Conclusions

Sodium tungsten bronze (Na-WO_3_) was synthesized hydrothermally and investigated by means of X-ray diffraction (XRD) and Fourier transform infrared (FTIR) spectroscopy. The obtained Na-WO_3_ materials were nanorods with typical diameters of 10–200 nm and lengths of several microns. The as-prepared Na-WO_3_ nanorods crystallized in a hexagonal structure (space group *P*6/*mmm*, No. 191) with unit cell parameters *a* = 7.3166(8) Å and *c* = 3.8990(8) Å and elongated along the ⟨001⟩ direction, representing a typical hexagonal tungsten oxide (HTO) material with interesting structural and physical properties. It was found that, during the electrochromic (EC) coloration, the proton insertion induced substantial changes in both long-range and local structural order, as evidenced by modified lattice parameters (*a* = 7.4192(6) Å, *c* = 7.5440(2) Å) and a new local symmetry (space group *P*6_3_/*mcm*, No. 193) for the EC colored Na-WO_3_ nanorods. The inserted protons were found to prefer the small trigonal windows over the larger trigonal cavities, suggesting a selective intercalation behavior analogous to that observed in the large hexagonal tunnels. FTIR results indicated that, during the bleaching process, the proton extraction in the small trigonal tunnels is closely associated with the dynamics of water molecules within the large hexagonal tunnels. Moreover, proton insertion and extraction are accompanied by electron transfer, leading to substantial modifications in the electronic structure of the material. Band gap narrowing (from 2.5 to 1.75 eV) and the appearance of a new near-infrared absorption band (centered at 1155 nm) were observed during the EC coloration, which can be attributed to increased free electron density resulting from the proton–electron double charge injection. These findings suggest that XRD, FTIR and other spectroscopic approaches should be more extensively employed to unravel the microscopic details of electrochromic mechanisms in HTOs and related materials.

## Data Availability

All relevant data during this study are included in this published article.
